# A protocol for a multi-site, spatially-referenced household survey in slum settings: methods for access, sampling frame construction, sampling, and field data collection

**DOI:** 10.1186/s12874-019-0732-x

**Published:** 2019-05-30

**Authors:** Improving Health in Slums Collaborative, Pauline Bakibinga, Caroline Kabaria, Catherine Kyobutungi, Anthony Manyara, Nelson Mbaya, Shukri Mohammed, Anne Njeri, Iqbal Azam, Romaina Iqbal, Shahida Mazaffar, Narjis Rizvi, Tayyaba Rizvi, Hamid ur Rehman, Syed A. K. Shifat Ahmed, Ornob Alam, Afreen Zaman Khan, Omar Rahman, Rita Yusuf, Doyin Odubanjo, Montunrayo Ayobola, Funke Fayehun, Akinyinka Omigbodun, Eme Owoaje, Olalekan Taiwo, Peter Diggle, Navneet Aujla, Yen-Fu Chen, Paramjit Gill, Frances Griffiths, Bronwyn Harris, Jason Madan, Richard J. Lilford, Oyinlola R. Oyobode, Vangelis Pitidis, Joao Porto de Albequerque, Jo Sartori, Celia Taylor, Philip Ulbrich, Olalekan Uthman, Samuel I. Watson, Godwin Yeboah

**Affiliations:** Improving Health in Slums Collaborative, Warwick Medical School, University of Warwick, Coventry, UK

**Keywords:** Survey, Slum, Sampling, GIS

## Abstract

**Background:**

Household surveys are a key epidemiological, medical, and social research method. In poor urban environments, such as slums, censuses can often be out-of-date or fail to record transient residents, maps may be incomplete, and access to sites can be limit, all of which prohibits obtaining an accurate sampling frame. This article describes a method to conduct a survey in slum settings in the context of the NIHR Global Health Research Unit on Improving Health in Slums project.

**Methods:**

We identify four key steps: obtaining site access, generation of a sampling frame, sampling, and field data collection. Stakeholder identification and engagement is required to negotiate access. A spatially-referenced sampling frame can be generated by: remote participatory mapping from satellite imagery; local participatory mapping and ground-truthing; and identification of all residents of each structure. We propose to use a spatially-regulated sampling method to ensure spatial coverage across the site. Finally, data collection using tablet devices and open-source software can be conducted using the generated sample and maps.

**Discussion:**

Slums are home to a growing population who face some of the highest burdens of disease yet who remain relatively understudied. Difficulties conducting surveys in these locations may explain this disparity. We propose a generalisable, scientifically valid method that is sustainable and ensures community engagement.

## Background

Slums are homes to a significant and growing proportion of the world’s population. It is estimated over one billion people will soon live in such areas worldwide, with almost all of these in low and middle income countries (LMICs) [1, 2]. The conditions that often define a slum, including a lack of access to clean water and sanitation, and of safe and durable housing, are also key risk factors for disease and poor welfare. Despite the increase in population and the significant health risks they face, slum residents remain an understudied population [2].

The NIHR Global Health Research Unit on Improving Health in Slums (the “Slum Health Project”) aims to examine the health care access and use, as well as to measure the health status, of slum residents in seven slum sites in four countries: Nigeria, Kenya, Pakistan and Bangladesh. A primary objective of the project is to complete a large-scale spatially-referenced survey of approximately 1000 households at each site. However, there is little guidance or consensus methodology on the conduct of such household surveys in complex urban areas like these, which frequently lack legal recognition, official censuses, or maps.

Household surveys are a major research tool used to capture data about a population’s socio-economic status, health status and behaviour, and other key characteristics. In order to conduct a survey, a sampling frame generally needs to be specified, i.e. the population from which the households are to be sampled. A complete sampling frame should list all possible households in the population of interest to ensure that the sampling method used can generate an unbiased sample. With a growing interest in spatial variation, geo-spatial analysis, and the consequences of spatial confounding (i.e. correlations in outcomes due to proximity rather than another shared cause of interest), an increasing number of household surveys are spatially referenced, i.e. the exact location of the household is recorded, and samples are designed to be spatially representative as opposed to completely random. As an example, the Demographic and Health Surveys (DHS) – a set of standardized, nationally representative surveys – are mostly spatially referenced [3]. The DHS and other similar surveys often use national censuses and the recorded population living in small enumeration areas or census tracts as a sampling frame. However, this may not be a sound basis for a sampling frame, particularly in the case of the slum context, for a number of reasons: (i) the information from a census may no longer be accurate with respect to where people reside if a significant amount of time has passed, for example, population turnover in two Nairobi slums was found to be approximately 25–30% of the population annually; [4] (ii) censuses can fail to cover people who are transient or who live in informal or makeshift accommodation; and (iii) censuses may not contain accurate location information, or census tracts may not be fine-grained enough to accurately represent the population distribution in very densely populated areas.

The slum context presents difficulties to conducting a valid household survey. This includes the aforementioned issues but also complex social and governmental relations that can complicate access. This may partially account for the disproportionately low level of research in these areas. In this article we propose a method to conduct a multi-site, spatially-referenced household survey in slum settings, which we illustrate with the specific example of the Slum Health Project. The study sites are seven slums across five cities in four countries: Lagos, Nigeria; Ibadan, Nigeria; Nairobi, Kenya; Karachi, Pakistan; and Dhaka, Bangladesh. We do not focus on the specific content of the surveys and the analysis of data generated from them, rather the aim of this protocol is to describe a generalisable method to produce a reliable and valid household survey in a slum setting, which can be replicated in new, similar settings. This includes accessing slum sites, constructing a spatially-referenced sampling frame, spatially-regulated sampling, and field data collection, management, and storage. The proposed method is intended to be relatively low-cost and sustainable, so that local residents may be equipped with the knowledge to continue to update maps, which in turn may be used to facilitate future research as well as for political recognition and planning.

## Methods/design

Both ‘household’ and ‘slum’ have multiple technical definitions. Different agencies define ‘slum’ in different ways. For example, UN Habitat specifies that a slum area is “any specific place, whether a whole city, or a neighbourhood, [...] if half or more of all households lack improved water, improved sanitation, sufficient living area, durable housing, secure tenure, or combinations thereof [5].” While UNESCO use: “A contiguous settlement where the inhabitants are characterised as having inadequate housing and basic services [6].” In our context, the study sites are well-known ‘slum’ areas whose boundaries are defined by the communities themselves or agreed among relevant stakeholders. Regardless of the definition, though, our method was designed to be applicable to any complex, irregular, or unplanned urban environment. For this article a necessary condition to be defined as a “household” was to be living in the same housing unit or connected premises. Other criteria such as having common cooking arrangements are frequently used, but are not required by this method.

The aims of the method described in this paper are to produce a valid spatially-regulated sample and to conduct a survey using this sample. Our sampling frame within each study-site is therefore the complete set of geo-located household locations. We describe firstly how access to slum sites might be obtained, secondly how a sampling frame is generated, thirdly how to sample from this sampling frame, and fourthly the process of collecting, managing and storing data from slum sites.

### Access to slum sites

Negotiating and obtaining access to conduct research in slum sites, which are often physically challenging and socially complex informal environments, requires an in-depth understanding of the political and social structures at local, community, and national levels. Slums are heterogeneous and the political and social structures within them vary between countries, cities, and even within the same city where more than one slum is present (e.g. [7, 8]).

The first step is a stakeholder mapping and engagement phase for each slum site. This is required in order to negotiate and obtain access, both from local authorities and local community leaders. The identification of all the relevant stakeholders (i.e. the “mapping”) should be undertaken by a local research team with knowledge of the national and local policy-drivers/makers as well as the political and social structures of the slum communities and local governments. There is not necessarily an optimal method to conduct the stakeholder mapping and engagement. A focus-group approach to stakeholder mapping can be cost-effective, rapid, and easily adaptable to a wide range of contexts. However, it may not always be suitable in practice as it is not always easy to bring together busy people. Similarly, political sensitivities or social hierarchies may affect group dynamics and ‘who’ speaks. Therefore, a combination of focus groups and one-on-one engagements may be more practical and appropriate.

The mapping and engagement exercise must consider each stakeholder’s interests and influences based upon their agenda, power-base, credibility, and consequences of the research for them, to ensure successful engagement. Understanding the sociology and political economy in slums is a key objective of stakeholder engagement exercises; however that aspect of the research is beyond the scope of this article, and here we focus on the issue of engaging stakeholders to negotiate access and site entry.

Table 1 provides some examples of access negotiations in the Slum Health Project. In all sites, we met with local community leaders and government officials, as well as NGOs and different community-based groups. The relevant government authority was the first point of contact followed by local community leaders. A “snowball” approach was taken, so that any additional stakeholders identified in the meetings were also engaged. Once access was negotiated, key stakeholders were kept appraised of all research activities, including times and dates when field workers would be present.

**Table 1:** Illustrative examples of negotiation of access to slum sites

*Kenya*	
The process for obtaining access to the two slum sites in Nairobi, Kenya firstly required engagement with the Nairobi City County Health Management team (HMT) to inform them of the planned research. A research protocol and ethical clearance letter from the nationally accredited Ethical Review Board was submitted. Following review, a research authorization letter was issued by the research committee copied to the relevant sub-county authorities. Pre-requisite authorization for the project was also obtained from the National Commission for Science, Technology and Innovation (NACOSTI). Subsequent meetings were held with the respective sub-county HMTs who made a recommendation to work with community health assistants who were conversant with different health service providers in the area. In addition, the local research team engaged with the local government chiefs based within the slum sites who arranged for meetings with Community Advisory Committees. The Community Advisory Committees comprising community leaders and representatives were briefed on the project objectives and given opportunity to air concerns regarding upcoming project activities. Access to the slum sites was granted during these meetings with CACs. Finally, an inception meeting was held in Nairobi to bring together county government officials, representatives from community advisory teams as well as NGO representatives in each slum site in order to explain the research project in more detail and how they can be involved as the research progresses.	
*Nigeria*	
Obtaining access to the three slum sites located in Ibadan and Lagos firstly required permission from the Governments of Oyo and Lagos States and to inform the chairpersons of the three relevant Local Government Areas (LGAs; the third-tier administrative unit). Once this permission was granted and advocacy visits had been made to the LGA chairpersons, researchers met with the local traditional leaders’ council of each of the communities. The site in Lagos had one traditional chief, while one in Ibadan had a committee of several local chiefs, with one selected by them as spokesperson. The remaining site in Ibadan has two local chiefs, one for the indigenous Yoruba community and another for the sizeable migrant Hausa ethnic group resident there. The study was explained to each traditional chief-in-council and their cooperation for the data collection exercises to be carried out in the communities was sought. Researchers also met with health practitioners operating within the study slum sites, which included traditional healers, patent medicine vendors, clinic matrons and proprietors of health facilities to ensure they were aware of the study, willing to provide information and welcomed researcher involvement. Lastly, in order to gain access to information about health facilities in the areas of study, permission was obtained from the State Ministry of Health and the Medical Officer of Health in each of the LGAs.	

### Constructing a sampling frame

**Fig. 1:**
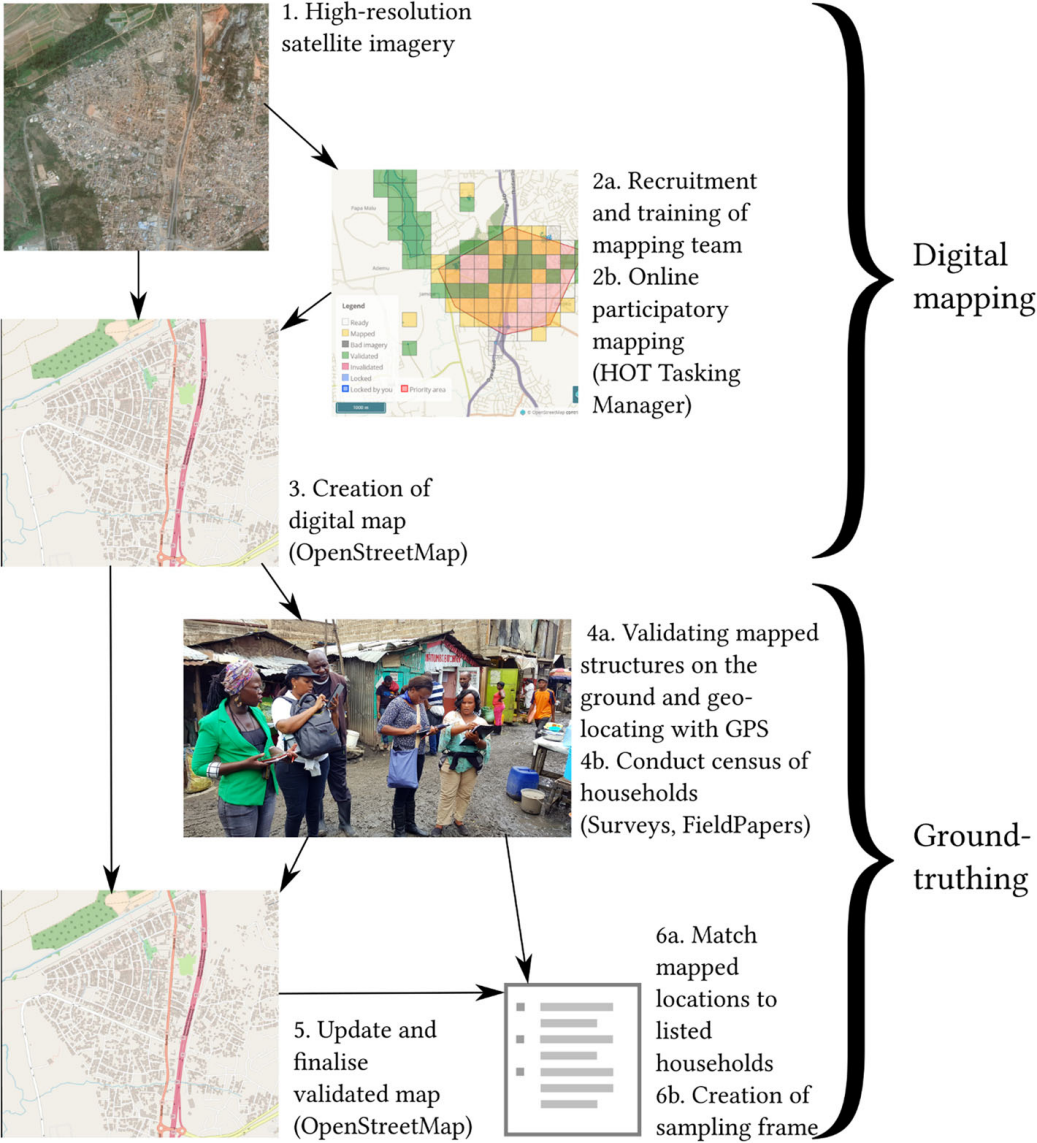
Flow diagram indicating mapping steps.© OpenStreetMap Contributors

In order to generate a spatially-regulated sample, the sampling frame must list all households in the area of interest and their precise locations. In high-income country settings, listings of households and their addresses are well-maintained, along with accurate detailed maps permitting the enumeration and geo-location of each household. Frequently, neither maps nor household listings exist for slums as the population is often not legally resident on the land, the structures that would be shown on maps are temporary and changeable, and there is little incentive for the state or private enterprise to produce maps. As a result there is a lack of information, formal or otherwise, about the location and function of structures and where households reside. Therefore, both a detailed map of all structures and a listing of households linked to locations on that map is required. Each country in the project formed a local mapping team, which included research staff and local community members and who were trained locally on each of the tasks described below. Figure 1 shows a simplified flow diagram of the processes to generate the sampling frame.

#### Generation of digital map data from satellite imagery

In this project, the slum boundaries were defined in collaboration with the local research team and slum community leaders. Official administrative or electoral boundaries can be used but can often be considered incorrect or out of date, particularly given the dynamic nature of the slum (e.g. [9]). Optical satellite images covering the study sites were procured from Airbus Intelligence at a resolution of ~ 30 cm – note that the resolution of freely available satellite imagery such as LandSat (~ 15 m) is insufficient for identifying the relevant features.

An online mapping platform was set up using the Humanitarian OpenStreetMap Team (HOT) Tasking Manager, which is a free online Geoweb infrastructure for coordinating remote participatory mapping, i.e. generation of map data from satellite imagery by a varied team in multiple locations (e.g. [10]). The HOT Tasking Manager subdivides the area of interest into smaller grids, each referred to as a “Task” that can be selected by a participant and mapped (Fig. 1, Step 2). Local project teams were first trained before recruiting additional participants including slum residents, OpenStreetMap communities (local and non-local), and other project team members. Once the digital maps are completed and validated against the satellite imagery, they need to be validated through “ground-truthing”, i.e. comparing the mapped features with observations on the ground. Each task is validated by an experienced mapper. The generated data are uploaded onto the OpenStreetMap online database (Fig. 1, Step 3) [11].

#### Onsite participatory mapping

The onsite participatory mapping is the “ground-truthing” stage: the accuracy of the digital map produced from the online mapping is checked in the field. This stage involves a number of steps. First, roads and footpaths are tracked in the study sites with handheld GPS devices to confirm their locations as mapped from the satellite imagery and produced in the digital map. Second, each structure is verified in two ways: if its geometry is incorrect, any changes are drawn on printed versions of the digital maps in the field, which are scanned and overlaid with the digital maps to make any corrections using the FieldPapers.org service; and third, each structure is surveyed using the digital data collection tools to generate unique identifiers for each structure, which are also marked indelibly on the structure for future identification, and to determine its function (e.g. residence or shop).

#### Identifying households

Where structures are identified as dwellings, each household as defined is recorded and identified by the name of the head of household or family name. High population turnover and lack of tenure or rental contract can result in households departing without notice. For the “Slum Health Project”, any household that had not been observed by neighbours for a period of 3 months or more is no longer considered ‘resident’. The spatial locations of the identified households are then linked to the relevant structure by the structure’s unique identifier. The location of the household is specified as the structure’s centroid. Once all structures are surveyed the sampling frame is complete.

### Sampling method

As slums typically exhibit very substantial spatial heterogeneity, it is desirable that the sampled locations span the whole of the site. A geometrically simple way to achieve this is to sample the households at, or as close as possible to, the points of a regular lattice overlaid on the mapped site. However, this has the disadvantage that it is biased in favour of sampling relatively isolated households. A completely random sample removes the bias but also results in uneven spatial coverage of the site. These considerations led Chipeta et al. [12] to suggest using an *inhibitory* sampling design, in which sampled locations are chosen at random subject to the spatially regulating constraint that no two sampled locations can be less than a specified distance *d* apart. The packing density of an inhibitory design is the fraction of the site area occupied by discs of diameter *d* centred on each sampled location. The maximum achievable packing density depends on the spatial arrangement of the available locations, here households, but in high-density settings a value of around 0.4 produces a highly regulated sample. Inhibitory designs are generally efficient for capturing spatial variation on the scale of the whole site, but cannot neither capture small-scale spatial variation nor distinguish it from non-spatial variation amongst the individuals who populate the sampled households. For this reason, Chipeta et al. [12] recommended tempering an inhibitory design by including a number of *close pairs*, i.e. augmenting an inhibitory design with a number of sampled locations, each one of which is located less than a specified distance *e* from the closest point of the inhibitory design, with *e* < *d*. In this context “close pairs” is taken to be households residing in the same structure (*e = 0)*. The number of close pairs and value of *d* will determined based on data from pilots conducted at each study site of between 20 and 30 households, which are purposively selected to maximise spatial variation.

Sample size in this context is often based on pragmatic considerations including resourcing and time. The “effective sample size” of a spatially correlated sample, i.e. the equivalently sized uncorrelated sample that provides the same information on a statistic of interest, is dependent on the degree of spatial correlation and the location of samples, among other things. Griffith [13] for example, provides a conceptual frame work for consider the effective sample size of spatially correlated samples.

### Data collection methods

Data collection methods in a slum context are dictated by similar considerations as any other context: data quality, data security (including relevant legislation such as the General Data Protection Regulation (GDPR) that came into force in the European Union in May 2018 [14]), ease of use, and costs. Based on the above criteria we recommend the use of digital tablet devices over paper-based forms as they reduce the risk of transcribing error, protect data security through encryption and not requiring the transport and storage of multiple paper forms, and reduces costs by not requiring extensive data entry. For the Slum Health Project digital tablet devices were purchased for all field workers and locked with a password. Field workers were required to sign agreements to use the tablets responsibility and all tablets are signed in and out.

**Fig. 2:**
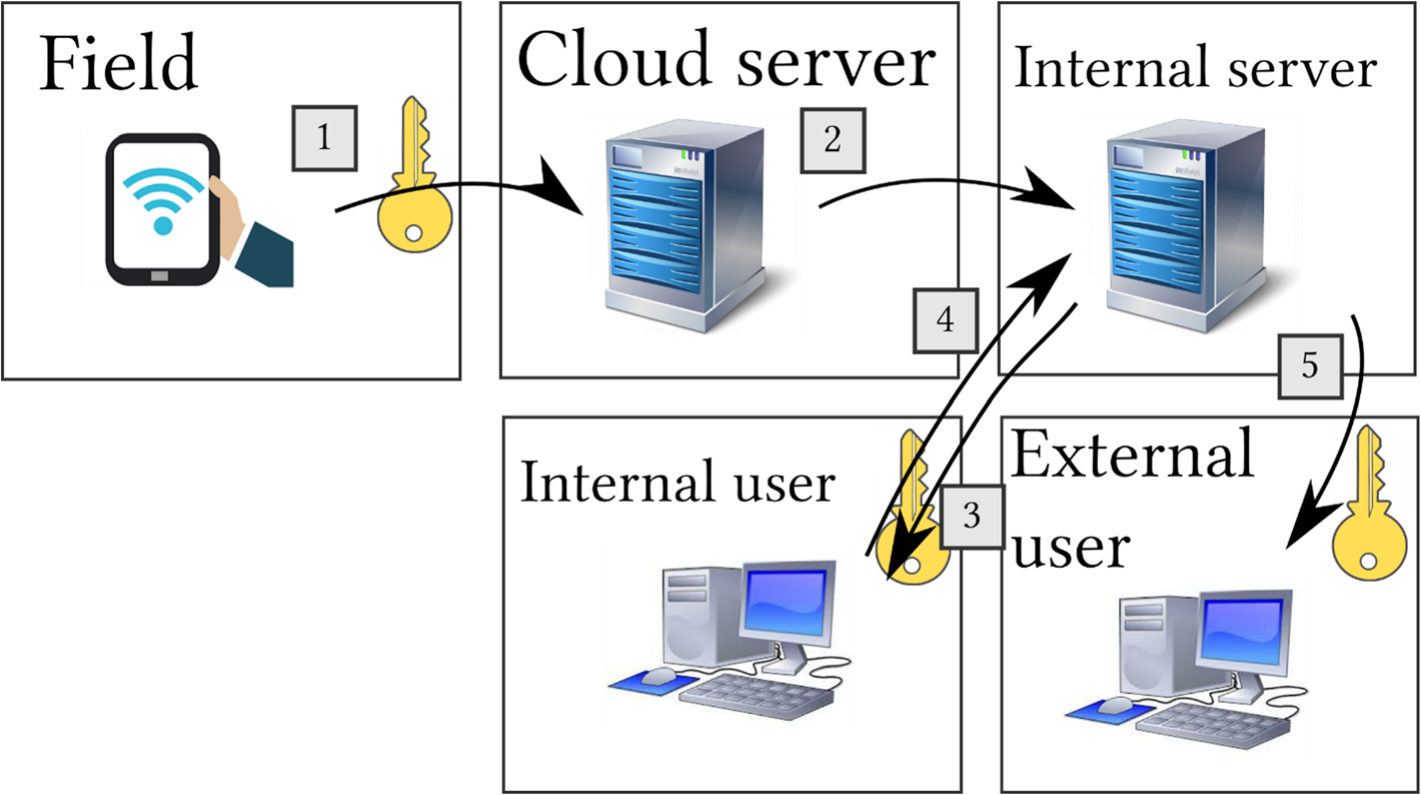
Data flows: (1) Validated field data are encrypted, submitted to the cloud server, and deleted from the tablet device. (2) Encrypted data are sent via SFTP to an internal storage server. (3) Data are downloaded, decrypted, for checking. (4) Processed data are re-encrypted and re-uploaded. (5) Authorised external users can download and decrypt the data. Key symbol represents encryption/decryption

In terms of software, a number of both proprietary and open-source options are available, including Open Data Kit, RedCap, and Survey CTO. We opted for the open-source Open Data Kit suite of software, which will improve sustainability [15]. Importantly this software permits offline data collection, automatic encryption, and uploads all submissions when the device is connected to the internet. Form programming was completed using *xlsform* [16]*.* These tools permit complex survey design and skip structures, can restrict responses to reduce errors, and collects locations, signatures, and images as required. A data aggregation server was set up using a (GDPR-compliant) cloud server provider that permits full control of the server and location of data storage to which access was strictly limited. Access to the server was secured using password-protected 256-bit SSH keys. A second data storage server was set up at the University of Warwick, to permit access to the data for project members and constitute a backup. The data collection process is as follows (Fig. 2):

1. Field workers conduct the interviews, completing the forms on the tablet devices. The responses are checked by a field supervisor for any potential errors. Additional quality control steps include spot checks by field supervisors, i.e. returning to a sampled house and re-asking a subset of questions, and sit-ins on interviews by supervisors. If any potential errors are identified, the field worker returns to confirm responses, otherwise the form is finalised. The software encrypts finalised forms using AES-256 encryption; following which forms are no longer accessible and are submitted automatically to the server when online and are deleted from the tablet (Fig. 2(1)).
2. Encrypted data are stored on the data aggregation server. When requested, the data are decrypted, the unique identifiers of submissions extracted and checked against a list of previous submissions, and if there are new submissions the data are processed (i.e. redundant columns removed, wide-form data converted to long-form, and separate data sets combined if they are from the same form, to facilitate use), and then re-encrypted using AES-256 encryption with a separate set of country-specific SSH keys. The data are then submitted to the data storage server via SFTP (Fig. 2(2)). Any unencrypted data are deleted.
3. Data are stored encrypted on the data storage server until required (Fig. 2(3) and (5)). Quality checks are conducted (Fig. 2(3) and (4)). On completion of data collection, the data will be ‘cleaned’ and merged into one final data set.

## Discussion

Conducting valid and reliable surveys in slum areas is a necessary but difficult undertaking. With few exceptions, such as the Map Kibera project [17] or the Nairobi Urban Health Demographic Surveillance System (NUHDSS) [18] both in Nairobi, Kenya, maps and censuses of slums are out-of-date or non-existent. However, a reliable sampling frame is necessary to select representative samples. Otherwise it is likely that the most transient or those not officially recognised as resident, who are also likely to have the highest levels of poverty and ill-health, may be missed. In this article we have detailed a method to create a spatially-referenced sampling frame consisting of a census of all households in a slum and thence a spatially-regulated representative sample. We discussed this in the context of a project investigating health care use and access in four countries. The method involves first generating a map using satellite imagery and then verifying and ground truthing this map. Once all structures are identified, their use is determined, and each household resident in each structure, if any, are identified. Households are located on the basis of the structure in which they reside. A spatially regulated sampling method is used. Digital data collection methods are also outlined.

There is no reliable means to validate these methods since no ‘Gold standard’ or even comparator data exists for these settings in general. However, each component of the overall method has a strong precedent. Participatory mapping using satellite imagery to generate maps quickly, cheaply, and reliably is widely used across many fields. For example, humanitarian response teams frequently use this technique in areas affected by natural disasters for planning [19, 20]. Ground-truthing to improve maps is also widely conducted and is considered necessary for valid maps. Ground truthing is an on-the-spot data gathering activity to verify what has already been collected in the past or remotely or to collect additional information. An example, in our context, is the Map Kibera project in Kenya where local residents mapped one of the largest slums in Africa to produce points of interest throughout the slum which eventually led to the GroundTruth Initiative [17, 21]. The advantage of these tools are that they are simple to use and the Open Street Map software is open source and online. Only a computer or smart phone is required for data input. This enables the continued maintenance of the maps generated in this project by local communities, providing an additional benefit of the work.

The growth of computing power has led to an increased ability to estimate complex statistical models that take account of spatial variation [22]. Observations are likely to be correlated with one another by virtue of their proximity, and not taking this into account may lead to biased estimates of population statistics or intervention effects. The spatial variation is also of interest in its own right for modelling disease prevalence and incidence and its relation to the environment [22]. Both of these types of analyses are particularly relevant in complex urban areas like slums which exhibit a high degree of spatial heterogeneity. Spatial-referencing is thus highly important and it is recommended for future work.

Obtaining access to slums is a key part of the methodology. Understanding the political and social contexts and obtaining buy in to the work from local stakeholders is key to the success of any research. For example, there is evidence to suggest people may misrepresent themselves if they believe doing so will result in benefits to their community [23]. Community engagement is therefore required to access the slums, ensure the reliability of the results, as well as build trust, improve communication, encourage feedback, and identify and respond to community need [24]. At the same time, the mapping, groundtruthing and survey processes we describe are “powerful tools in and of themselves for community engagement [24].”

We acknowledge there may be weaknesses to the methods discussed here. Populations living in slums can be highly mobile. Similarly makeshift structures are liable to be demolished and rebuilt. This may render the maps incorrect over a relatively short time scale. The validity and usefulness of these methods are therefore closely entwined with their sustainability: only by providing the community the training and tools to update and maintain their maps can it be ensured they will remain accurate after any project funding has ended.

This article discusses methods for conducting a spatially-referenced household survey in slum areas, which are applicable to other complex urban settings. Much of the method that has been proposed mirrors that of household surveys in other urban areas, however, in general previous household surveys have been conducted where there are reliable sampling frames. This method builds on recent programme-specific efforts to map slum-based households (e.g. [17]): conducting surveys in slums requires flexibility and contextualisation in light of the mapping and research history of the site of interest and key stakeholders. Slum surveys remain rare despite the large and growing population of slum dwellers, who also carry the highest burden of disease and poverty. These methods provide a key example for future work in this area.

## Abbreviations

DHS: Demographic and health survey
GDPR: General data protection regulation
GIS: Geographic information systems
LMIC: Low and middle income country
NGO: Non-governmental organisation
NIHR: National Institute of Health Research
NUHDSS: Nairobi Urban Health Demographic Surveillance System
